# Health Risks of Sarcopenic Obesity in Overweight Children and Adolescents: Data from the CHILT III Programme (Cologne)

**DOI:** 10.3390/jcm11010277

**Published:** 2022-01-05

**Authors:** Carolin Sack, Nina Ferrari, David Friesen, Fabiola Haas, Marlen Klaudius, Lisa Schmidt, Gabriel Torbahn, Hagen Wulff, Christine Joisten

**Affiliations:** 1 Department for Physical Activity in Public Health, Institute of Movement and Neurosciences, Am Sportpark Müngersdorf 6, German Sport University Cologne, 50933 Cologne, Germany; nina.ferrari@uk-koeln.de (N.F.); d.friesen@dshs-koeln.de (D.F.); f.haas@dshs-koeln.de (F.H.); m.klaudius@dshs-koeln.de (M.K.); ernaehrungschmidt@gmail.com (L.S.); c.joisten@dshs-koeln.de (C.J.); 2 Cologne Center for Prevention in Childhood and Youth/Heart Center Cologne, University Hospital of Cologne, Kerpener Str. 62, 50937 Cologne, Germany; 3 Department of Pediatrics, Paracelsus Medical University, Breslauer Str. 201, 90471 Nuremberg, Germany; gabriel.torbahn@klinikum-nuernberg.de; 4 Department of Sport and Health Sciences, Faculty of Human Sciences, University of Potsdam, Karl-Liebknecht-Str. 24–25, 14476 Potsdam, Germany; hagen.wulff@uni-potsdam.de

**Keywords:** sarcopenia, sarcopenic obesity, muscle-to-fat ratio, juvenile obesity

## Abstract

Sarcopenic obesity is increasingly found in youth, but its health consequences remain unclear. Therefore, we studied the prevalence of sarcopenia and its association with cardiometabolic risk factors as well as muscular and cardiorespiratory fitness using data from the German Children’s Health InterventionaL Trial (CHILT III) programme. In addition to anthropometric data and blood pressure, muscle and fat mass were determined with bioelectrical impedance analysis. Sarcopenia was classified via muscle-to-fat ratio. A fasting blood sample was taken, muscular fitness was determined using the standing long jump, and cardiorespiratory fitness was determined using bicycle ergometry. Of the 119 obese participants included in the analysis (47.1% female, mean age 12.2 years), 83 (69.7%) had sarcopenia. Affected individuals had higher gamma-glutamyl transferase, higher glutamate pyruvate transaminase, higher high-sensitivity C-reactive protein, higher diastolic blood pressure, and lower muscular and cardiorespiratory fitness (each *p* < 0.05) compared to participants who were ‘only’ obese. No differences were found in other parameters. In our study, sarcopenic obesity was associated with various disorders in children and adolescents. However, the clinical value must be tested with larger samples and reference populations to develop a unique definition and appropriate methods in terms of identification but also related preventive or therapeutic approaches.

## 1. Introduction

Obesity in children and adolescents is a growing health problem [1]. Between 1975 and 2016, the global prevalence of overweight in children and adolescents worldwide increased from 0.7% to 5.6% in girls and 0.9% to 7.8% in boys [2]. In Germany, the prevalence of overweight including obesity was 15.4% among children aged 3–17 years based on the Child and Adolescent Health Survey (KiGGS, wave 2 2014–2017) [1]. The current COVID-19 pandemic is expected to lead to a further increase [3].

In addition to the possible persistence of overweight and obesity into adulthood, along with its corresponding health consequences [4], the significantly increased risk of cardiometabolic, orthopaedic, and psychological comorbidities is problematic even in this young age group [5,6]. Children and adolescents with overweight and obesity are more likely to have cardiovascular risk factors such as high blood pressure, lipid metabolism disorders, and glucose metabolism disorders. This may lead to the development of non-communicable diseases such as type 2 diabetes mellitus [1,7,8], endothelial dysfunction, non-alcoholic fatty liver disease (NAFLD), and musculoskeletal dysfunction [1,6,9]. The complete picture of the metabolic syndrome (MetS) is found in 6% to 39% of overweight children, depending on the underlying definition [10]. Visceral fat content, the associated secretion of so-called adipocytokines [11], and the presence of low-threshold systemic inflammation play a central role in the development of the above-mentioned comorbidities [12]. In addition, an inverse relationship between cardiometabolic risk factors and low or disproportionate (with respect to body fat) muscle mass is increasingly being described in adulthood as expression of the so-called sarcopenic obesity [13,14,15]. Classically, sarcopenia is associated with underweight due to the loss of muscle mass as well as reduced muscle strength and function. The European Working Group on Sarcopenia in Older People (EWGSOP) expanded the definition of sarcopenia to include both primary sarcopenia–characterised by reduced muscle mass, limited muscle function, and strength at an older age and secondary sarcopenia in the context of chronic diseases, including obesity [16]. However, people with sarcopenic obesity can be of normal weight or ‘only’ overweight, but their relatively low muscle mass may be masked by a higher fat mass [14,15,17]. Thus, in addition to measuring handgrip strength, the muscle-to-fat ratio (MFR) is used to determine the severity of sarcopenic obesity [17,18,19]. MFR is an indicator for cardiometabolic risk factors and metabolic syndrome in adults, while it correlates negatively with waist circumference, systolic blood pressure, and blood lipid levels [19]. Additionally, MFR can also be used to assess cardiometabolic health in children [20].

Despite these initial indications, few studies to date have examined the presence of sarcopenic obesity in childhood and adolescence or possible concomitant diseases [17,18]. In order to avoid the negative health consequences of sarcopenic obesity, adequate knowledge and evidence-informed countermeasures are urgently needed. Therefore, we investigated the correlations between the presence of cardiometabolic risk factors and the occurrence of sarcopenia/sarcopenic obesity using the Children’s Health Interventional Trial (CHILT III) programme, which is an outpatient weight management programme for obese children and their families.

## 2. Materials and Methods

### 2.1. Sample Description

In this study, the input data of the CHILT III programme of the German Sport University Cologne from the years 2003–2021 were used, which is a family-based, multimodal, outpatient programme for obese children and adolescents aged between 8 and 16 years [21].

**Figure 1:**
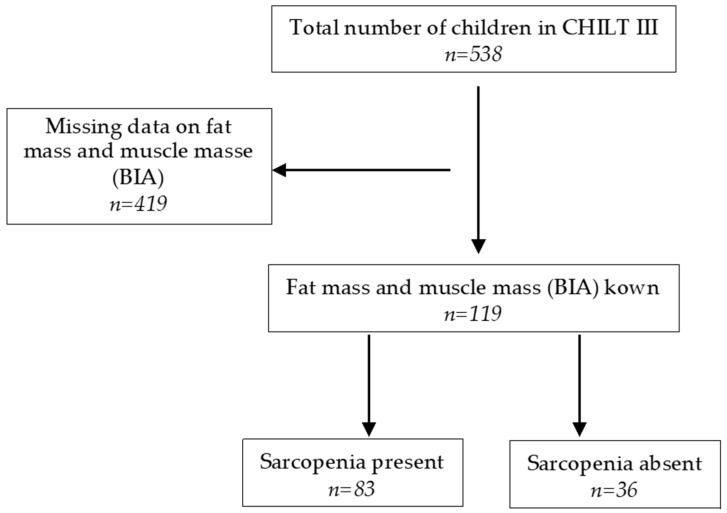
Flowchart of the selected subjects.

Of 538 subjects, 119 (47.1% female) could be included in the analysis, as it was possible to classify sarcopenia by MFR followed by Kim et al. [22]. The MFR cut-off values were defined according to McCarthy et al. [20] (cut-off = mean value − 2SD of the MFR of the middle fifth of the BMI range). Based on this, the MFR cut-off value for sarcopenia is at 1.25 for boys of all ages, 1.1 for girls between 5–10 years, and 0.8 for girls between 10–18 years [20]. Accordingly, the diagnosis of sarcopenia was made when the MFR was below these cut-off values. Therefore, in 83 children (69.7%), sarcopenia was present according to the above criteria (see Figure 1). None of them suffered from an overt diabetes mellitus type I or II.

#### 2.1.1. Anthropometric Data

The height of the children and adolescents was measured barefoot in cm; weight was measured in kg. A calibrated scale and a stadiometer were used for this purpose [23]. BMI was divided into percentiles according to Kromeyer-Hauschild et al. [24]. Following the guidelines of the ‘Arbeitsgemeinschaft für Adipositas (AGA)’, a BMI above the 90th percentile was classified as overweight and a BMI above the 97th percentile was classified as obese [25]. In addition, the BMI standard deviation score (SDS) was calculated using the least mean squares (LMS) method for non-normally distributed characteristics [25]:

(1) $${\mathrm{SDS}}_{\mathrm{LMS}}=\frac{{\left[\mathrm{BMI}/M[t]\right]}^{L[t]}-1}{L[t]S[t]}$$

M[t], L[t], and S[t] are parameters for the participants’ age and sex.

Waist circumference was measured in cm using a standard tape measure. The measurement was taken midway between the anterior superior iliac spine and the lowest rib with the participant standing upright. Using a body fat caliper (Harpender Skinfold Caliper HSK-BI, British Indicators, West Sussex, UK), skinfold thickness was measured in triplicate to the nearest 0.2 mm in triceps and subscapular according to a standardised protocol [26], and the mean of the three results was reported. Sex- and age-specific equations were used to calculate body fat percentage, as in similar studies [27,28,29].

#### 2.1.2. Bioelectrical Impedance Analysis

Fat mass and muscle mass were determined by bioelectrical impedance analysis (BIA; Nutriguard-MS, Data Input GmbH, Pöcking, Germany). This was determined to 0.1 kg and 0.1% with the four-point measurement. The frequency of the measurement was 50 kHz [30]. The following values were determined from the measurement: resistance (^®^), reactance (Xc), checksum (Σ), total resistance (Rtot.), and phase angle (φ). Using the NutriPlus software (NutriPlus, Data Input GmbH, Pöcking, Germany), fat and muscle mass in kg were calculated from these values and reported [31].

#### 2.1.3. Skeletal Muscle Mass, Fat Mass, and Muscle-to-Fat Ratio

In addition, skeletal muscle mass (SMM) was calculated according to Janssen et al. [32]:

SMM (kg) = [Ht^2^/R × 0.401) + (sex × 3.825) + (age × −0.071)] + 5.102 (2)

(Ht = height in cm, R = BIA − resistance in Ω, sex = 1 = male, 0 = female, age in years).

This formula assumes a strong correlation between SMM as calculated by MRI and BIA resistance [32]. Furthermore, the skeletal muscle index (SMI) in % (SMM/body mass × 100) was calculated [33].

The SMI was also calculated according to Park et al. [13]:

SMI (kg/m^2^) = SMM (kg)/body size (m^2^). (3)

The MFR was calculated according to McCarthy et al. [20]:

MFR = SMM in kg/body fat mass (FM) in kg.(4)

For the calculation of the MFR, in this study, skeletal muscle mass and fat mass were given and used by the NutriPlus software of BIA measurements [31].

#### 2.1.4. Blood Pressure

Blood pressure was measured oscillometrically three times using an automatic blood pressure monitor after approximately 5–10 min of rest [23]. The cuff size was chosen so that two-thirds of the upper arm length was covered. The mean value from all three calculations was calculated and documented. We classified hypertension according to the S2k guideline of the German Society for Paediatric Cardiology using age- and height-specific reference values [34].

#### 2.1.5. Laboratory Parameters

Blood values were taken after fasting (12 h food and drink abstinence, including plain water, no teeth brushing) and analysed in the laboratory of the German Sport University. Fasting blood glucose, total cholesterol, high-density lipoprotein (HDL), and triglycerides were measured directly. Low-density lipoprotein (LDL) cholesterol was determined indirectly from total cholesterol, HDL, and triglycerides using the Friedewald equation [35]. Insulin was determined using human insulin standards (Elecsys Insulin) from Roche Diagnostics, Mannheim [36]. The homeostatic model assessment (HOMA index) was used as a parameter of insulin sensitivity and was calculated by the following formula [23,37]:

HOMA = (insulin [mU/L] × glucose [mmol/L])/22.5.(5)

In addition, GGT (in U/L) was determined by a kinetic photometric assay using reagents from ABX Pentra (HORIBA ABX 2007). GPT and GOT (each in U/L) were determined via an optimised UV assay without pyridoxal phosphate using reagents from ABX Pentra (HORIBA ABX 2005/2007) [36]. High-sensitivity (hs) CRP (in mg/L) was determined using the cobas c system by Roche/Hitachi. Leptin was measured by a direct sandwich enzyme-linked immunosorbent assay (ELISA, kit from MERCK/Millipore KgaA, Darmstadt, Germany).

#### 2.1.6. Definition of Metabolic Syndrome (MetS)

The metabolic syndrome was defined according to the International Diabetes Federation (IDF) classification modified for children and adolescents up to 16 years [38]. For adolescents over 16 years, we applied the IDF criteria for adults [39] (see Appendix A Table A1).

#### 2.1.7. Cardiorespiratory Fitness/Ergometry

Maximum cardiorespiratory performance capacity (in watts) was determined by bicycle ergometry (Ergometrics er900, Ergoline, Bitz, Germany). The children started at 25 watts, and workload was increased by 25 every 2 min until the maximum load was reached. The children were encouraged to continue until they had reached their maximum physical capacity. Relative watts (watts/kg) was defined as the maximum watts in relation to body weight [21].

Children with acute illnesses, such as febrile infections, asthma attacks, or metabolic diseases were excluded from ergometry. Other contraindications include cardiomyopathies, certain vascular anomalies, and heart failure [40].

#### 2.1.8. Muscular Fitness/Standing Long Jump

The standing long jump was used to measure muscular fitness, resp. jumping strength, and associated leg muscle strength. This test was based on the Dordel–Koch test with defined standard values. In the test, the children and adolescents had to jump as far as possible using both legs without a run-up. The children made two attempts, and the better one was scored [41].

### 2.2. Statistical Analysis

The data were analysed using IBM SPSS Statistics, version 28.0; descriptive statistics were presented as means and standard deviations (SD). Normal distribution of the parameters was checked using the Kolmogorov–Smirnov test. Parameters with normal distribution were examined with parametric tests. Parameters that were not normally distributed were tested using the Mann–Whitney U-test. A *t*-test was used to compare the means of continuous parameters with variance homogeneity. In the case of variance heterogeneity, the T-test with Welch correction was used. We tested categorical parameters using a Chi-squared test. Statistical significance was defined as a *p*-value < 0.05.

## 3. Results

### 3.1. Anthropometry

Table 1 shows the anthropometric data of the total sample, as well as the possible differences between participants with and without sarcopenia based on the above-mentioned cut-offs. Of the children with sarcopenia (*n* = 83), 69.9% were male and 30.1% were female; in the group without sarcopenia, (*n* = 36), 13.9% were male and 86.1% were female (*p* < 0.001). On average, children and adolescents with and without sarcopenia were the same age, height, and weight (see Table 1). However, there were significant differences in BMI-SDS (*p* = 0.018) and waist circumference (*p* = 0.024).

In children with sarcopenia, boys had a higher waist circumference (*p* = 0.042), higher GPT (*p* = 0.006), higher skeletal muscle mass, and higher SMI (*p* < 0.001), as well as a higher MFR (*p* = 0.026) than girls (see Appendix A Table A2, Table A3 and Table A4).

### 3.2. Laboratory Parameters and Blood Pressure

Children with sarcopenia showed a significantly higher GGT (*p* = 0.028), higher GPT (*p* = 0.003), a significantly higher hs-CRP (*p* = 0.009), and significantly higher diastolic blood pressure (*p* = 0.046). The other parameters did not differ significantly among participants with and without sarcopenia (see Table 2).

### 3.3. Muscle Mass and Cardiorespiratory/Muscular Fitness

Children with sarcopenia had higher BIA fat mass (*p* = 0.032) and lower MFR (*p* < 0.001), lower physical performance (*p* = 0.001), and lower jumping distance (*p* = 0.041). Muscle mass, SMM, and SMI did not differ significantly (see Table 3).

### 3.4. Sarcopenia and MetS

Regarding the classification of the metabolic syndrome according to the modified IDF classification [38] (see Appendix A Table A1) and the presence of sarcopenia, the Chi-squared test showed no significant difference (*p* = 0.747).

## 4. Discussion

To our knowledge, this is one of the first studies to examine sarcopenia in overweight and obese children and adolescents and its association with selected cardiometabolic risk factors and exercise capacity. Of the participants, 69.7% were characterised as sarcopenic, which was associated with higher values for waist circumference, BMI-SDS, GGT, GPT, hs-CRP, and diastolic blood pressure, and lower cardiorespiratory and muscular fitness. There was no correlation between sarcopenia and systolic blood pressure, lipids, or fasting blood glucose, insulin levels, and HOMA index, or components of the MetS. However, already in this age group, indications of systemic inflammation, NAFLD, and blood pressure are shown.

It may be possible that the disease value would have been clearer with a larger and less homogeneous sample (all the children and adolescents were obese). So far, the occurrence of sarcopenia or sarcopenic obesity has been analysed mainly in the context of older and/or chronically ill subjects [42]. Orkin et al. showed that BIA measures of muscle and fat mass correlate strongly with magnetic resonance imaging (MRI) measures of total psoas muscle surface area (tPMSA) and fat areas in children with obesity and NAFLD. However, they did not explicitly take into account the occurrence of sarcopenia [43].

As with older persons, there is a lack of gold standard for the definition of sarcopenic obesity in children and adolescents [22,42]. We followed the cut-off values of the MFR (cut off = mean value − 2SD of the MFR of the middle fifth of BMI range) defined by McCarthy et al. using BIA muscle and fat mass [20]. Further studies using dual energy X-ray (DEXA) for body composition showed that with a lower cut-off value (cut-off value of the mean value minus 1 SD of the MFR for the third BMI quintile), the proportion of children below this value is higher [22]. Therefore, it must be critically questioned whether sarcopenic obesity is adequately represented by the ratio of muscle to fat mass. However, in addition to the MFR, other methods for determining muscle strength, mass, and power are recommended for the diagnosis of sarcopenia [16].

While DEXA serves as the gold standard for determining muscle mass, it is significantly more time consuming and expensive than BIA. Segmental BIA has proven to be an effective and practical alternative [16,20,44] especially when using multi-frequency devices as in our analysis [30]. Chen et al. compared the results of BIA and DEXA in 1476 children and adolescents aged 7–17 years and showed high comparability in the determination of body fat [45]. Similar studies used the appendicular skeletal muscle mass (the sum of the skeletal muscle mass in all four extremities) to calculate muscle mass by Tanita BC-418MA single frequency (50 Hz) Segmental Body Composition Analyser [20].

Additionally, muscle strength in general is measured by handgrip strength [16]. Due to a lack of data on handgrip strength, we analysed the results of the standing long jump to determine muscular fitness in the lower limbs, as other studies have shown that this is a valid parameter [46,47].

In older persons, muscle performance is often determined by the short physical performance battery, the 6-min walk, or the timed get-up-and-go test [16]. For our younger population, we added cardiorespiratory fitness measured in watts/kg.

However, taking into account the methodological approach, this cross-sectional analysis shows that sarcopenic obesity in children and adolescents increases the risk of systemic inflammation or NAFLD, diastolic blood pressure, and poorer cardiorespiratory and muscular fitness. In association with other inflammatory cytokines, adipokines and/or myokines such as IL-6 and TNF-alpha should be examined in larger collectives. However, first of all, a unique definition and assessment in this age group has to be developed. Additionally, future prospective studies should consider measuring the clinical significance of sarcopenia and sarcopenic obesity in children and adolescence. In addition to general primary prevention measures in kindergartens and schools to promote a healthy lifestyle (including the preservation of muscle mass), we recommend including parameters such as MFR in paediatric health examinations.

This study has strengths and limitations in addition to those already mentioned. One strength is the presence of factors relevant to the assessment of sarcopenic obesity and possible associated disorders. However, in this rather small, selected group, the pubertal status was not taken into account, because the Tanner stage was not recorded. Studies have shown that puberty can reduce the risk of elevated total cholesterol and LDL cholesterol [48]. In addition, puberty involves a physiological insulin resistance of the body, which is a central element in the development of a MetS [11]. Another limitation is the use of BIA to determine body compositions as mentioned above. A determination of the body compositions by DEXA and on this basis determined could possibly lead to more precise results [17]. For further studies, especially in the development of a uniform definition of sarcopenic obesity in this age group, the use of a DEXA is recommended.

As a main limitation, we have already pointed out the lack of a clear definition and methodical recording of sarcopenia in children and adolescents. The laboratory determination of GGT and GPT does not necessarily mean that NAFLD is present. Abdominal ultrasound examinations or even liver biopsies were not possible in our study. In addition to the determination of the MFR, simpler and more accessible methods for diagnosing sarcopenia should be implemented. One suggestion is the handgrip-to-BMI ratio defined by Steffl et al. [17].

Lower relative handgrip strength in children was associated with higher BMI and waist circumference [18]. Therefore, the authors recommended using the handgrip to BMI ratio to identify children at a risk of sarcopenic obesity [17]. The extent to which this would lead to different results remains speculative at present.

## 5. Conclusions

In summary, sarcopenia according to the used definition is present in more than two-thirds of our population of children and adolescents with overweight and obesity. In this group, sarcopenic obesity is associated with poorer cardiorespiratory and muscular fitness, elevated GGT, GPT, and hs-CRP levels, and elevated diastolic blood pressure. However, to identify the clinical value, a unique definition and methods not only based on the ration between muscle and fat mass to identify children at risk are preconditions. Subsequently, appropriate preventive and therapeutic countermeasures at an early stage have to be developed.

## Figures and Tables

**Table 1:** Anthropometric data for the whole group and by the presence of sarcopenia.

Parameter	Total	Sarcopenia Absent	Sarcopenia Present	*p*-Value
Age (years)	12.2 ± 2.2 (*n* = 119)	12.4 ± 1.9 (*n* = 36)	12.2 ± 2.3 (*n* = 83)	*p* = 0.635 ^†^
Height (m)	1.57 ± 0.12 (*n* = 118)	1.58 ± 0.08 (*n* = 35)	1.57 ± 0.14 (*n* = 83)	*p* = 0.623 ^†^
Weight (kg)	76.6 ± 22.8 (*n* = 118)	73.0 ± 16.7 (*n* = 35)	78.1 ± 24.9 (*n* = 83)	*p* = 0.469 ^‡^
BMI (kg/m^2^)	30.4 ± 5.6 (*n* = 118)	29.0 ± 4.3 (*n* = 35)	31.0 ± 6.0 (*n* = 83)	*p* = 0.083 ^‡^
BMI-SDS	2.52 ± 0.48 (*n* = 118)	2.36 ± 0.44 (*n* = 35)	2.58 ± 0.48 (*n* = 83)	*p* = 0.018 ^†^*
Waist circumference (cm)	96.2 ± 14.8 (*n* = 117)	91.1 ± 11.2 (*n* = 35)	98.3 ± 15.6 (*n* = 82)	*p* = 0.024 ^‡^*

Data are presented as mean ± SD (*n* = number). Abbreviations: BMI = body mass index; SDS = standard deviation score. ^†^ *t*-test for presence of sarcopenia, ^‡^ Mann–Whitney U-test, * statistical significance.

**Table 2:** Blood pressure and laboratory parameters of the whole group and by the presence of sarcopenia.

Parameter	Total	Sarcopenia Absent	Sarcopenia Present	*p*-Value
Systolic blood pressure (mmHg)	115.2 ± 12.1 (*n* = 119)	114.6 ± 12.0 (*n* = 36)	115.5 ± 12.2 (*n* = 83)	*p* = 0.720 ^‡^
Diastolic blood pressure (mmHg)	70.8 ± 8.1 (*n* = 119)	68.5 ± 7.6 (*n* = 36)	71.8 ± 8.1 (*n* = 83)	*p* = 0.046 ^‡^*
Fasting blood glucose (mg/dL)	92.2 ± 7.1 (*n* = 95)	92.5 ± 8.2 (*n* = 30)	92.1 ± 6.5 (*n* = 65)	*p* = 0.779 ^†^
Insulin (µU/mL)	24.4 ± 11.7 (*n* = 86)	25.7 ± 11.8 (*n* = 24)	23.9 ± 11.7 (*n* = 62)	*p* = 0.516 ^‡^
HOMA Index	5.6 ± 2.7 (*n* = 86)	5.9 ± 2.7 (*n* = 24)	5.5 ± 2.7 (*n* = 62)	*p* = 0.441 ^‡^
Total cholesterol (mg/dL)	165.4 ± 26.8 (*n* = 96)	160.9 ± 21.3 (*n* = 30)	167.5 ± 28.9 (*n* = 66)	*p* = 0.268 ^†^
HDL cholesterol (mg/dL)	45.4 ± 8.7 (*n* = 96)	44.1 ± 8.9 (*n* = 30)	45.9 ± 8.6 (*n* = 66)	*p* = 0.354 ^†^
LDL cholesterol (mg/dL)	97.7 ± 24.3 (*n* = 96)	94.5 ± 19.8 (*n* = 30)	99.1 ± 26.1 (*n* = 66)	*p* = 0.388 ^†^
Triglycerides (mg/dL)	119.2 ± 69.9 (*n* = 96)	118.6 ± 51.9 (*n* = 30)	119.4 ± 77.1 (*n* = 66)	*p* = 0.543 ^‡^
GOT (U/L)	27.4 ± 14.9 (*n* = 95)	24.4 ± 7.9 (*n* = 30)	28.8 ± 17.1 (*n* = 65)	*p* = 0.207 ^‡^
GPT (U/L)	29.2 ± 30.5.(*n* = 95)	21.1 ± 14.3 (*n* = 30)	33.0 ± 35.1 (*n* = 65)	*p* = 0.003 ^‡^*
GGT (U/L)	23.8 ± 13.5 (*n* = 93)	19.7 ± 5.5 (*n* = 29)	25.6 ± 15.5 (*n* = 64)	*p* = 0.028 ^‡^*
hs-CRP (mg/L)	3.7 ± 3.0 (*n* = 30)	1.4 ± 1.3 (*n* = 6)	4.3 ± 3.0 (*n* = 24)	*p* = 0.009 ^‡^*
Leptin (ng/mL)	11.6 ± 6.2 (*n* = 28)	15.7 ± 11.2 (*n* = 5)	10.7 ± 4.4 (*n* = 23)	*p* = 0.684 ^‡^

Data are presented as mean ± SD (*n* = number). Abbreviations: HOMA = homeostasis model assessment; HDL = high-density lipoprotein; LDL = low-density lipoprotein; GOT = glutamate oxaloacetate transaminase; GPT = glutamate pyruvate transaminase; GGT = gamma-glutamyl transferase; hs-CRP = high-sensitivity C-reactive protein. ^†^ *t*-test for the presence of sarcopenia, ^‡^ Mann–Whitney U-test, * statistical significance.

**Table 3:** SMM, SMI, MFR, cardiorespiratory, and muscular fitness of the whole group and by the presence of sarcopenia.

Parameter	Total	Sarcopenia Absent	Sarcopenia Present	*p*-Value
Fat mass (kg), anthropometric	19.8 ± 7.0 (*n* = 119)	18.2 ± 4.9 (*n* = 36)	20.5 ± 7.7 (*n* = 83)	*p* = 0.214 ^‡^
Fat mass (kg), BIA	28.1 ± 10.6 (*n* = 119)	24.5 ± 7.7 (*n* = 36)	29.6 ± 11.4 (*n* = 83)	*p* = 0.032 ^‡^*
Muscle mass (kg), BIA	23.6 ± 7.4 (*n* = 119)	24.3 ± 5.8 (*n* = 36)	23.3 ± 7.9 (*n* = 83)	*p* = 0.163 ^‡^
SMM (kg)	24.8 ± 5.6 (*n* = 118)	23.7 ± 4.4 (*n* = 35)	25.2 ± 6.0 (*n* = 83)	*p* = 0.242 ^‡^
SMI (%)	33.3 ± 5.2 (*n* = 118)	33.1 ± 4.5 (*n* = 35)	33.4 ± 5.5 (*n* = 83)	*p* = 0.748 ^‡^*
SMI (kg/m^2^)	9.4 ± 1.8 (*n* = 118)	9.7 ± 1.5 (*n* = 35)	9.2 ± 1.9 (*n* = 83)	*p* = 0.074 ^‡^
MFR	0.89 ± 0.22 (*n* = 119)	1.04 ± 0.23 (*n* = 36)	0.82 ± 0.18 (*n* = 83)	*p* < 0.001 ^‡^*
Relative cardiorespiratory fitness (watts/kg)	1.6 ± 0.5 (*n* = 118)	1.9 ± 0.4 (*n* = 36)	1.6 ± 0.5 (*n* = 82)	*p* = 0.001 ^†^*
Muscular fitness/standing long jump (cm)	108.8 ± 22.9 (*n* = 84)	116.2 ± 22.4 (*n* = 27)	105.3 ± 22.5 (*n* = 57)	*p* = 0.041 ^†^*

Data are presented as mean ± SD (*n* = number). Abbreviations: BIA = bioelectric impedance analysis; SMM = skeletal muscle mass; SMI = skeletal muscle index; MFR = muscle-to-fat ratio. ^†^ *t*-test for the presence of sarcopenia, ^‡^ Mann–Whitney U-test, * statistical significance.

## Data Availability

The data used and analysed during the current study involve sensitive patient information and indirect identifiers. As a result, the datasets are not available.

## Appendix A

Table A1: Definitions of the metabolic syndrome, Table A2: Anthropometric data for the group of sarcopenia present in girls and boys, Table A3: Blood pressure and laboratory parameters of the group of sarcopenia present in girls and boys, Table A4: SMM, SMI, MFR, cardiorespiratory fitness, and standing long jump of the group of sarcopenia present in girls and boys.

**Table A1:** Definitions of the metabolic syndrome.

Modified IDF Definition [38] for 10–16-Years Old	IDF Definition > 16 Years Old [39]
WC ≥ 90. percentile	WC m ≥ 94 cm, f ≥ 80 cm or BMI ≥ 30 kg/m^2^
SBP ≥ 130 mmHg or DBP ≥ 85 mmHg	SBP ≥ 130 mmHg or DBP ≥ 85 mmHg or treatment of previously diagnosed hypertension
Triglycerides ≥ 150 mg/dL (≥1.7 mmol/L)	Triglycerides ≥ 150 mg/dL or specific treatment for this lipid abnormality
HDL < 40 mg/dL (<1.03 mmol/L)	HDL m < 40 mg/dL (<1.03 mmol/L), f <50 mg/dL (<1.29 mmol/L)
Fasting blood glucose ≥ 100 mg/dL (≥5.6 mmol/L) or DM type 2	Fasting blood glucose ≥100 mg/dL (≥5.6 mmol/L) or DM type 2
MetS = WC ≥ 90. percentile + 2 criteria	MetS = WC m ≥ 94 cm, f ≥ 80 cm or BMI ≥ 30 kg/m^2^ + 2 criteria

Abbreviations: WC = waist circumference; SPB = systolic blood pressure; DPB = diastolic blood pressure; HDL = high-density lipoprotein; DM = diabetes mellitus; m = men; w = women.

**Table A2:** Anthropometric data for the group of sarcopenia present in girls and boys.

Parameter	Girls	Boys	*p*-Value
Age (years)	11.6 ± 2.5 (*n* = 25)	12.4 ± 2.2 (*n* = 58)	*p* = 0.209 ^‡^
Height (m)	1.53 ± 0.13 (*n* = 25)	1.58 ± 0.14 (*n* = 58)	*p* = 0.105 ^‡^
Weight (kg)	72.1 ± 20.8 (*n* = 25)	80.7 ± 26.2 (*n* = 58)	*p* = 0.288 ^‡^
BMI (kg/m^2^)	30.1 ± 5.0 (*n* = 25)	31.4 ± 6.3 (*n* = 58)	*p* = 0.481 ^‡^
BMI-SDS	2.62 ± 0.44 (*n* = 25)	2.57 ± 0.49 (*n* = 58)	*p* = 0.627 ^‡^
Waist circumference (cm)	92.4 ± 9.7 (*n* = 25)	100.9 ± 17.0 (*n* = 57)	*p* = 0.042 ^‡^*

Data are presented as mean ± SD (*n* = number). Abbreviations: BMI = body mass index; SDS = standard deviation score. ^‡^ Mann–Whitney U-test for girls/boys, * statistical significance.

**Table A3:** Blood pressure and laboratory parameters of the group of sarcopenia present in girls and boys.

Parameter	Girls	Boys	*p*-Value
Systolic blood pressure (mmHg)	113.2 ± 11.9 (*n* = 25)	116.6 ± 12.3 (*n* = 58)	*p* = 0.136 ^‡^
Diastolic blood pressure (mmHg)	73.5 ± 7.3 (*n* = 25)	71.0 ± 8.4 (*n* = 58)	*p* = 0.164 ^‡^
Fasting blood glucose (mg/dL)	89.8 ± 5.4 (*n* = 19)	93.0 ± 6.8 (*n* = 46)	*p* = 0.079 ^‡^
Insulin (µU/mL)	22.1 ± 11.0 (*n* = 17)	24.5 ± 12.1 (*n* = 45)	*p* = 0.390 ^‡^
HOMA Index	5.0 ± 2.7 (*n* = 17)	5.6 ± 2.7 (*n* = 45)	*p* = 0.273 ^‡^
Total cholesterol (mg/dL)	171.7 ± 27.2 (*n* = 19)	165.8 ± 29.7 (*n* = 47)	*p* = 0.332 ^‡^
HDL cholesterol (mg/dL)	47.6 ± 6.3 (*n* = 19)	45.3 ± 9.4 (*n* = 47)	*p* = 0.272 ^‡^
LDL cholesterol (mg/dL)	102.9 ± 23.7 (*n* = 19)	97.6 ± 27.1 (*n* = 47)	*p* = 0.506 ^‡^
Triglycerides (mg/dL)	109.8 ± 52.5 (*n* = 19)	123.3 ± 85.2 (*n* = 47)	*p* = 0.745 ^‡^
GOT (U/L)	23.9 ± 8.1 (*n* = 19)	30.9 ± 19.3 (*n* = 46)	*p* = 0.063 ^‡^
GPT (U/L)	21.5 ± 12.4 (*n* = 19)	37.7 ± 40.1 (*n* = 46)	*p* = 0.006 ^‡^*
GGT (U/L)	23.4 ± 16.2 (*n* = 18)	26.5 ± 15.4 (*n* = 46)	*p* = 0.175 ^‡^
hs-CRP (mg/L)	3.9 ± 2.5 (*n* = 7)	4.4 ± 3.3 (*n* = 17)	*p* = 0.852 ^‡^
Leptin (ng/mL)	9.1 ± 2.9 (*n* = 7)	11.4 ± 4.8 (*n* = 16)	*p* = 0.308 ^‡^

Data are presented as mean ± SD (*n* = number). Abbreviations: HOMA = homeostasis model assessment; HDL = high-density lipoprotein; LDL = low-density lipoprotein; GOT = glutamate oxaloacetate transaminase; GPT = glutamate pyruvate transaminase; GGT = gamma-glutamyl transferase; hs-CRP = high-sensitivity C-reactive protein. ^‡^ Mann–Whitney U-test for girls/boys, * statistical significance.

**Table A4:** SMM, SMI, MFR, cardiorespiratory fitness, and standing long jump of the group of sarcopenia present in girls and boys.

Parameter	Girls	Boys	*p*-Value
Fat mass (kg), anthropometric	18.7 ± 6.3 (*n* = 25)	21.3 ± 8.1 (*n* = 58)	*p* = 0.258 ^‡^
Fat mass (kg), BIA	28.5 ± 9.8 (*n* = 25)	30.1 ± 12.1 (*n* = 58)	*p* = 0.770 ^‡^
Muscle mass (kg), BIA	21.0 ± 5.8 (*n* = 25)	24.3 ± 8.6 (*n* = 58)	*p* = 0.130 ^‡^
SMM (kg)	20.2 ± 3.6 (*n* = 25)	27.4 ± 5.5 (*n* = 58)	*p* < 0.001 ^‡^*
SMI (%) ^1^	29.0 ± 4.1 (*n* = 25)	35.3 ± 5.0 (*n* = 58)	*p* < 0.001 ^‡^*
SMI (kg/m^2^)	8.8 ± 1.4 (*n* = 25)	9.4 ± 2.1 (*n* = 58)	*p* = 0.202 ^‡^
MFR	0.76 ± 0.11 (*n* = 25)	0.85 ± 0.19 (*n* = 58)	*p* = 0.026 ^‡^*
Relative cardiorespiratory fitness (watts/kg)	1.6 ± 0.6 (*n* = 24)	1.5 ± 0.4 (*n* = 58)	*p* = 0.717 ^‡^
Muscular fitness/standing long jump (cm)	100.2 ± 9.8 (*n* = 16)	107.2 ± 25.7 (*n* = 41)	*p* = 0.248 ^‡^

Data are presented as mean ± SD (n = number). Abbreviations: BIA = bioelectric impedance analysis; SMM = skeletal muscle mass; SMI = skeletal muscle index; MFR = muscle-to-fat ratio. ^‡^ Mann–Whitney U-test girls/boys, * statistical significance, ^1^ Janssen et al. [33].
